# Recent advances in managing and understanding uveitis

**DOI:** 10.12688/f1000research.10587.1

**Published:** 2017-03-16

**Authors:** Shih-Chou Chen, Shwu-Jiuan Sheu

**Affiliations:** 1 Department of Ophthalmology, Kaohsiung Veterans General Hospital, Kaohsiung, Taiwan; 2 School of Medicine, National Yang-Ming University, Taipei, Taiwan

**Keywords:** immunosuppressive therapy, ocular implants, optical coherence tomography (OCT), uveitis

## Abstract

Uveitis is a sight-threatening disease entity with intraocular inflammation that arises from various causes. It mainly affects working-age individuals and may lead to irreversible visual loss if not treated properly in a timely manner. This article reviews recent advances in the management and understanding of uveitis since 2014, including treatment with new immunosuppressive therapies that use biological agents, local therapy with steroid implants, and imaging studies for the evaluation of uveitis.

## Introduction

Uveitis is a sight-threatening disease entity with intraocular inflammation that arises from various causes. It may lead to irreversible visual loss if not treated properly in a timely manner. As many as 35% of patients with uveitis exhibit blindness or visual impairment in at least one eye ^1^. Uveitis is the fifth most common cause of severe visual loss in the developed world, and up to 20% of legal blindness is due to complications of uveitis ^2, 3^.

On the basis of etiology, uveitis can be divided into infectious uveitis and non-infectious uveitis. In infectious uveitis, treatments are aimed mainly at the pathogens. In non-infectious uveitis, systemic corticosteroids are the most widespread treatment regimen. Because of the undesirable side effects and restricted function of systemic corticosteroids, the use of immunosuppressants and local therapy with steroid implants has become more popular in recent years.

Many advances have been made in the management and understanding of uveitis in the 3 years between 2014 and 2016. In this review, we briefly discuss uveitis treatment with new immunosuppressive therapies based on biological agents and local therapy with steroid implants; many of these novel approaches are currently being evaluated in clinical trials. New progress in imaging studies for the evaluation of uveitis, including enhanced depth imaging (EDI), optical coherence tomography (OCT), and ultra-wide-field fluorescein angiography, will also be discussed.

## Biological agents

Advances in molecular research have confirmed that the dysregulation of inflammatory cytokines plays an important role in the pathogenesis of immune-mediated disease and have allowed the development of new targeted therapies that interfere with specific molecules that cause inflammation and tissue damage ^4^. New targeted therapies or biological agents are widely used in rheumatology and, in recent years, have been introduced into the management of refractory uveitis. Biological agents, including cytokine inhibitors, monoclonal antibodies, and growth factor inhibitors, have been made using recombinant DNA technology. Many clinical studies about the development and outcome of new biological agents for uveitis treatment have been published in recent years. In addition, various types of intravitreal injections have been tried, and the results have been published. Targeted delivery of therapeutic agents is expected to decrease the need for systemic therapy, and to reduce the associated side effects, but not to replace systemic treatment.

### Adalimumab

Adalimumab is a human anti–tumor necrosis factor-alpha (anti–TNF-α) monoclonal antibody. It was the first fully human monoclonal antibody drug approved by the US Food and Drug Administration (FDA). Adalimumab and infliximab were the best-studied anti–TNF agents in ophthalmology and indeed have now been recommended by an expert panel for first-line treatment of ocular manifestations of Behçet’s disease and second-line treatment of other forms of uveitis ^5, 6^.

Several studies about adalimumab in the treatment of uveitis were published recently. For example, Jaffe *et al.* conducted a multinational phase 3 trial that involved 217 patients who had active non-infectious intermediate uveitis, posterior uveitis, or panuveitis ^7^. Patients were randomly assigned to receive adalimumab or matched placebo. Outcomes regarding change in anterior chamber cell grade, vitreous haze grade, and best corrected visual acuity (BCVA) were significantly better in the adalimumab group than in the placebo group. However, adverse events and serious adverse events were reported more frequently among patients who received adalimumab. Van Denderen *et al.* demonstrated the effect of systemic adalimumab in treating acute uveitis secondary to ankylosing spondylitis in 77 patients ^8^. A significant reduction in the number of anterior uveitis attacks per patient was observed during adalimumab treatment.

Cordero-Coma *et al.* evaluated the immunogenicity induced by adalimumab on the basis of drug serum level and clinical responses in 25 patients with non-infectious uveitis who were resistant to conventional therapy ^9^. Median trough adalimumab serum levels were higher in responders than in non-responders. Development of permanent antibodies against adalimumab was associated with undetectable trough adalimumab levels and worse uveitis outcomes in non-responders. The short-term efficacy of intravitreal adalimumab (IVA) for the treatment of eyes with active non-infectious uveitis was reported by Hamam *et al.* ^10^. Six patients (12 eyes) completed 26 weeks of IVA treatment. Seven eyes had improvement of at least two Early Treatment Diabetic Retinopathy Study (ETDRS) lines, nine out of ten eyes with vitreous haze had no haze at 26 weeks, and five out of eight eyes with macular edema had complete resolution. IVA was effective in controlling inflammation, decreasing macular edema, and improving BCVA in the majority of eyes in this series.

### Infliximab

Infliximab is a chimeric anti–TNF-α monoclonal antibody used to treat autoimmune diseases. An expert panel also recommended infliximab as a first-line treatment of ocular manifestations of Behçet’s disease and second-line treatment for other forms of uveitis ^5, 6^.

The efficacy of adalimumab and infliximab in refractory uveitis due to Behçet’s disease was described by Calvo-Río *et al.* ^11^. In 124 patients with Behçet’s disease uveitis refractory to conventional treatment, including high-dose corticosteroids and at least one standard immunosuppressive agent, 77 patients received infliximab and 47 patients received adalimumab. In the majority of patients, a decrease in anterior chamber and vitreous inflammation, a reduction in macular thickness, and improvements in BCVA were observed.

Takeuchi *et al.* conducted a multicenter study to evaluate the long-term efficacy and safety of infliximab treatment in 164 Behçet’s disease patients with uveitis ^12^. Infliximab reduced the frequency of ocular attacks, improved visual acuity, and was generally well tolerated with few serious adverse events. Approximately 80% of relapses occurred within 1 year after the initiation of infliximab treatment, and 90% of these relapses were controlled by increasing doses of topical corticosteroids and shortening the interval between infliximab infusions.

Kruh *et al.* conducted a retrospective chart review to analyze the safety and efficacy of infliximab for the treatment of refractory non-infectious uveitis ^13^. Of the 88 patients with chronic and recalcitrant uveitis treated with infliximab, 72 patients (81.8%) achieved clinical remission. Thirty-two patients (36.4%) experienced at least one side effect, and 17 patients (19.3%) discontinued treatment because of intolerable side effects. The most common adverse effects were skin rash (9.1%) and fatigue (8%). Infliximab induces a high rate of complete clinical remission in recalcitrant uveitis and is well tolerated by most patients.

### Etanercept

Etanercept is a fusion protein that acts as a TNF inhibitor and is used in the treatment of rheumatoid arthritis, juvenile idiopathic arthritis (JIA), psoriatic arthritis, plaque psoriasis, and ankylosing spondylitis. Saeed *et al.* reported about five patients with JIA-related uveitis who previously received methotrexate but had suboptimal responses in controlling uveitis and were switched to etanercept treatment ^14^. Three out of five patients did not show any signs of uveitis at their last follow-up. Etanercept may be useful in controlling JIA-related uveitis or arthritis in pediatric patients when methotrexate has had a suboptimal effect in controlling the inflammatory activity. Most experts will agree that etanercept is not a good option for the management of uveitis but is a good option for arthritis. There are a few reports linking this agent to inducing episodes of uveitis.

### Golimumab

Golimumab is a human IgG1 TNF-α antagonist monoclonal antibody and is approved by the FDA for once-monthly subcutaneous administration as a treatment for rheumatoid arthritis, psoriatic arthritis, ankylosing spondylitis, and ulcerative colitis. The affinity of golimumab for soluble human TNF-α, as determined by surface plasmon resonance, was similar to that of etanercept, greater than that of infliximab, and significantly greater than that of adalimumab ^15^.

Miserocchi *et al.* reported the long-term efficacy of subcutaneous injections of golimumab 50 mg monthly in 17 patients (34 eyes) with severe recalcitrant uveitis who had inadequate response to previous biologics ^16^. Of the 17 patients, 14 patients responded to golimumab. Visual acuity remained stable in 26 eyes, improved in 7, and worsened in 1. This study concluded that golimumab may be a promising new therapeutic option for severe uveitis patients who have not responded to other biologics. Similar results were reported in 3 patients with non-infectious uveitis that was refractory to standard immunosuppressive drugs and had previously been treated with other anti–TNF-α drugs ^17^.

### Secukinumab

Secukinumab is a fully human anti–interleukin-17A monoclonal antibody that has been approved by the FDA for the treatment of ankylosing spondylitis and psoriatic arthritis. Letko *et al.* conducted a multicenter randomized clinical trial to evaluate the efficacy and safety of secukinumab in 37 patients with non-infectious uveitis ^18^. Intravenous 30 mg/kg secukinumab produced higher response rates (72.7% versus 33.3%) and remission rates (27.3% versus 16.7%) compared with subcutaneous 300 mg secukinumab ^18^. Intravenous secukinumab was effective and well tolerated in non-infectious uveitis that required systemic corticosteroid-sparing immunosuppressive therapy. High-dose intravenous secukinumab may be necessary to deliver secukinumab in therapeutic concentrations.

### Tocilizumab

Tocilizumab is a humanized monoclonal antibody against the interleukin-6 receptor and is used mainly for the treatment of rheumatoid arthritis and JIA. Papo *et al.* observed eight patients with severe and refractory non-infectious uveitis treated with tocilizumab 8 mg/kg every 4 weeks intravenously ^19^. Seven patients had been previously treated with anti–TNF-α agents. The immunosuppressive drugs used in association with tocilizumab were azathioprine (n = 2), mycophenolate mofetil (n = 2), and methotrexate (n = 2). After a median follow-up of 8 months, six patients improved after tocilizumab treatment and two patients were non-responders. Visual acuity improved in five patients. The long-term effects of tocilizumab therapy for refractory uveitis-related macular edema was supported by a study by Mesquida *et al.* ^20^. According to this case series, tocilizumab seems to be a safe and promising therapy in severe and refractory non-infectious uveitis.

## Other immunosuppressive therapies

### Sirolimus

Sirolimus (previously known as rapamycin) is produced by a strain of *Streptomyces hygroscopicus*. Sirolimus is a potent inhibitor of the antigen-induced proliferation of T cells, B cells, and antibodies. Demonstration of the potent immunosuppressive activity of sirolimus in animal models of organ transplantation led to clinical trials and subsequent approval by the FDA for prophylaxis against renal graft rejection ^21^.

Early phase I/II studies have provided encouraging safety and efficacy data concerning the use of sirolimus to treat uveitis. Sirolimus appears to be an important addition to the armamentarium of steroid-sparing therapeutic agents that act on various steps in the inflammatory pathway ^22^. Phase III clinical trials are ongoing. Intravitreal sirolimus appears to be an interesting option for the treatment of non-infectious posterior uveitis because of its highly targeted molecular effects, non-steroidal nature, and good safety profile ^23^.

### Intravenous immunoglobulin therapy

Intravenous immunoglobulin (IVIG) therapy involves the use of immunoglobulin mixtures. It has multiple mechanisms of immunomodulatory action and is used to treat various autoimmune, infectious, and idiopathic diseases. IVIG is approved for the treatment of autoimmune idiopathic thrombocytopenic purpura, allogeneic bone marrow transplantation, Kawasaki disease, and chronic lymphocytic leukemia.

According to a retrospective chart review of four patients with active non-infectious uveitis refractory to steroids and immunomodulatory therapy, three patients who received IVIG experienced disease stabilization and prevention of disease progression. But treatment failed to induce long-term remission in one patient who experienced a recurrence of macular edema ^24^.

## Corticosteroid implants

Corticosteroids are the first-line therapy in the treatment of most cases of uveitis. Topical corticosteroids are effective in the treatment of anterior uveitis but provide only poor response in intermediate uveitis, posterior uveitis, and panuveitis because of the drug’s limited diffusion into target tissues. Periocular injection improves the drug delivery, but the outcome is still not satisfactory. Systemic administration of corticosteroids affords higher drug concentration but leads to undesirable side effects such as increased body weight, puffy face, gastric ulcers, hyperglycemia, hypertension, insomnia, osteoporosis, and growth retardation in children, among other undesirable effects.

Intravitreal triamcinolone had been used off-label for quite some time, and its effects on the control of inflammation and reduction of macular edema are well documented but entail significant side effects on intraocular pressure and cataract formation ^25^. Park *et al.* treated 49 eyes with intravitreal triamcinolone acetonide injection for refractory Behçet’s posterior uveitis ^26^. The patients’ mean BCVA scores improved from 0.89 logMAR units to 0.70 and 0.64 at 12 and 24 months, respectively, and complete inflammation control was achieved in 87% of patients. But ocular complications, including cataract formation and elevated intraocular pressure, limit the efficacy and repeatability of this treatment.

Intravitreal injection of a slow-release corticosteroid implant offers better safety and efficacy for the treatment of non-infectious intermediate or posterior uveitis. Currently available steroid implants indicated for uveitis include Retisert (0.59 mg fluocinolone acetonide; Bausch & Lomb, Rochester, NY, USA) and Ozurdex (700 μg dexamethasone; Allergan, Irvine, CA, USA). In a phase III study (the Chronic Uveitis Evaluation of the Intravitreal Dexamethasone Implant (HURON **)** clinical trial), a single dexamethasone intravitreal implant significantly reduced intraocular inflammation and improved visual acuity for 6 months in patients with non-infectious intermediate or posterior uveitis. The incidence of elevated intraocular pressure and cataract formation did not differ significantly from that of the placebo group in this short-term study ^27^. In a multicenter study by Zarranz-Ventura *et al.*, Ozurdex implants were associated with favorable visual acuity and vitreous haze scores, but the treatment requires repeated injections ^28^. The cumulative effects in improving retinal thickness and resolving ocular inflammation were shown in a retrospective long-term series that also showed minimal complications ^29^. Complications included cataract progression and elevated intraocular pressure; the latter was mostly controllable by pressure-lowering agents. The reported complication rates were lower than those associated with Retisert or triamcinolone.

### Corticosteroid implants and pediatric uveitis

Bratton *et al.* reported that dexamethasone implants, in combination with systemic immunomodulatory therapy, resulted in improved visual acuity, control of intraocular inflammation, and a decrease in corticosteroid use during the treatment of pediatric uveitis ^30^. The uveitis reoccurred in 57% of eyes at 4.3 months (range of 2–7 months) after injection. The adverse events were similar to those identified in adult studies. These effects were supported by other series that showed that repeated implantations resulted in continued control of the inflammation, allowing a reduction of systemic immunosuppression with few ocular complications ^31, 32^. However, more data are required to establish the long-term safety of the implants in the pediatric age group.

### Multicenter Uveitis Steroid Treatment trial

The Multicenter Uveitis Steroid Treatment (MUST) trial identified 479 intermediate, posterior, and panuveitic eyes randomly assigned to either systemic immunosuppression or intravitreal fluocinolone acetonide implant therapy ^33^. Similarly favorable 2-year outcomes following both systemic treatment and fluocinolone acetonide implant treatment were found. Eyes that presented with more prolonged or severe inflammatory findings initially or during follow-up had a worse visual prognosis ^33^. The results at 54 months showed that visual outcomes after fluocinolone acetonide implant and systemic treatment for intermediate uveitis, posterior uveitis, and panuveitis were similar ^34^. For bilateral uveitis cases, systemic treatment may be necessary as the initial treatment on the basis of the cost-effectiveness. But steroid implant therapy can be considered in unilateral uveitis cases and when systemic treatment is not successful ^34^.

The risk and quality-of-life (QoL) analysis of the MUST trial at 54-month follow-up revealed an association between fluocinolone acetonide implant therapy and increased risk of glaucoma and cataracts. In self-reported QoL analysis, implant therapy was favored in initial time. But similar QoL results were disclosed between implant therapy and systemic therapy groups over time ^35^.

### Corticosteroid implants and Behçet’s posterior uveitis

Coşkun *et al.* investigated 17 eyes of 12 patients with active Behçet’s posterior uveitis that received a single intravitreal injection of a dexamethasone implant ^36^. The mean BCVA was significantly increased, the mean central macular thickness was significantly decreased, and the vitreous haze score was significantly decreased from baseline at each control visit at months 1, 3, 6, and 12. Three eyes showed spikes of intraocular pressure that required topical anti-glaucoma treatment ^36^.

### Review of corticosteroid implants

Burkholder *et al.* conducted an anonymous online survey to describe the practice patterns and perceptions of uveitis specialists regarding the use of intravitreal dexamethasone implants for the treatment of non-infectious uveitis ^37^. The authors found no clear consensus on preferences regarding the use of dexamethasone versus fluocinolone acetonide implants.

Brady *et al.* conducted a search of the Cochrane database about the use of corticosteroid implants to treat chronic non-infectious uveitis ^38^. The study included randomized controlled trials comparing either fluocinolone acetonide or dexamethasone intravitreal implants versus standard-of-care therapy with at least 6 months of follow-up. Two studies compared fluocinolone acetonide implants with standard-of-care therapy and showed that fluocinolone acetonide implants probably prevent recurrence of uveitis compared with standard-of-care therapy (risk ratio of 0.29), but the implants were associated with increased risks of needing cataract surgery (risk ratio of 2.98) and surgery to lower intraocular pressure (risk ratio of 7.48). No studies have compared dexamethasone implants with standard-of-care therapy ^38^.

## Imaging studies in uveitis

### Enhanced depth imaging–optical coherence tomography in uveitis

Enhanced depth imaging–optical coherence tomography (EDI-OCT) was able to image the choroid with reasonable clarity using commercial spectral-domain OCT and proved to be a promising novel technique for imaging the choroid ^39^. With the introduction of EDI, visualization of the choroid and choriocapillaris has become possible. Therefore, OCT has become an indispensable ancillary test in the diagnosis and management of inflammatory diseases involving the retina and choroid ^40^.

Yan *et al.* investigated the retinal and choroidal thickness in 148 eyes from 97 patients with inactive uveitis and 98 eyes from 55 normal patients using spectral-domain OCT with EDI of the retina and choroid ^41^. The mean subfoveal retinal thickness did not differ significantly between uveitis patients and controls. The mean choroidal thickness at multiple locations was significantly lower in uveitis patients compared with normal patients; this difference was most significant at the fovea. The choroidal thickness was reduced in patients with inactive uveitis and was associated with disease duration and frequency, although retinal thickness did not seem to be affected by disease processes.

Macular edema is a common cause of visual loss in patients with uveitis. Géhl *et al.* used spectral-domain OCT with EDI scans to evaluate the retinal and choroidal thickness of 21 patients with anterior uveitis, 23 patients with intermediate uveitis, and 34 age-matched healthy controls ^42^. The mean central retinal subfield thickness was significantly higher in the intermediate uveitis group but was not significantly different in the anterior uveitis group compared with the control group. The retina in both uveitis groups was significantly thicker in the 3- and 6-mm perifoveal rings than those in the control group, but no significant difference was found in central choroidal thickness.

### Anterior segment–optical coherence tomography in uveitis

Anterior segment–OCT (AS–OCT) was developed specifically for imaging anterior segment structures. Sharma *et al.* designed an observational case series to determine the feasibility of AS–OCT to objectively image and quantify the degree of anterior chamber inflammation in patients with uveitis ^43^. In the 76 patients with 114 eyes collected, 83 eyes were imaged with line scans and 31 eyes were imaged with volume scans. The number of cells detected with line scans and volume scans was correlated with clinical grading. Automated algorithm had a strong correlation with manual measurement when measuring cell counts in three-dimensional volume scans. AS–OCT can be used to measure anterior chamber inflammation to identify responses to treatment in patients with uveitis ^43^.

### Evaluation of vitreous inflammation by optical coherence tomography

OCT plays an important role in the evaluation of vitreous inflammation intensity. Keane *et al.* collected 30 eyes of 30 patients with vitreous haze secondary to intermediate, posterior, or panuveitis; 12 eyes of 12 patients with uveitis without vitreous haze; and 18 eyes of 18 patients without intraocular inflammation ^44^. The severity of vitreous haze was classified on the basis of the National Eye Institute system. Spectral-domain OCT was used to measure vitreous (VIT) signal intensity and compared with intensity of retinal pigment epithelium (RPE), generating a ratio of “VIT/RPE-relative intensity”. This ratio was significantly higher in uveitic eyes than in uveitic eyes without vitreous haze or in healthy controls. The VIT/RPE-relative intensity showed a significant, positive correlation with clinical vitreous haze scores ^44^.

The same result of significant positive correlation with vitreous haze score and VIT/RPE-relative intensity was demonstrated in a retrospective cohort study by Zarranz-Ventura *et al.* with 105 uveitic eyes of 105 patients, which remained significant after adjusting for factors affecting media clarity such as anterior chamber cells, flares, and phakic status ^45^. Sreekantam *et al.* also used spectral-domain OCT to evaluate the vitreous inflammation in patients with uveitic cystoid macular edema before and after sub-Tenon’s triamcinolone acetonide injection ^46^. Treatment with triamcinolone acetonide resulted in a significant reduction in VIT/RPE-relative intensity, central retinal thickness, and improvement in visual acuity. These results provide evidence that OCT is useful in measurements of vitreous intensity and outcome in patients with uveitis.

### Ultra-wide-field fluorescein angiography

The peripheral retina is the site of pathology in many ocular diseases, and ultra-wide-field imaging is one of the new technologies available for ophthalmologists as they manage these diseases. With the advent of ultra-wide-field fluorescein angiography, it is now possible to view up to 200° of the retina in a single photograph measured from the ocular center ^47^.

Karampelas *et al.* investigated the relationships between peripheral vasculitis, ischemia, and vascular leakage in 82 uveitis patients by using ultra-wide-field fluorescein angiography ^48^. Although central leakage was associated with peripheral leakage, there was no association between foveal avascular zone size and peripheral ischemia. Peripheral ischemia correlated with neovascularization-related leakage and focal vasculitis. Only macular ischemia and increased macular thickness were independently associated with reduced visual acuity.

Chi *et al.* evaluated peripheral retinal vascular changes in anterior uveitis by using ultra-wide-field fluorescein angiography in 65 eyes of 33 patients with anterior uveitis ^49^. Peripheral vessel leakage was detected in 27 eyes (42%) with anterior uveitis, of which 15 eyes displayed active inflammation and 12 eyes displayed inactive inflammation. Peripheral vessel leakage was found in seven out of eight eyes with cystoid macular edema. The treatment strategies for these patients were modified on the basis of the results of ultra-wide-field fluorescein angiography ^49^.

## Summary

The diagnosis and treatment of uveitis have advanced markedly in recent years. We reviewed publications regarding the management and understanding of uveitis during the past three years, including biological agents, steroid implants, and imaging studies for the evaluation and treatment of uveitis. The progress in imaging techniques helps physicians understand the pathogenesis of uveitis and can also be used for the diagnosis and follow-up of disease activity or as a useful guide to treatment. Recent and ongoing studies are contributing to more focused treatments, including biologic agents and intraocular implants.
